# Research and Outcome Priorities for the Canada Prenatal Nutrition Program in Ontario: A Community‐Engaged Modified Delphi Study

**DOI:** 10.1111/mcn.70257

**Published:** 2026-10-15

**Authors:** Jo‐Anna B. Baxter, Alison Mildon, Bronwyn Underhill, Daniel W. Sellen, Deborah L. O'Connor

**Affiliations:** ^1^ Department of Nutritional Sciences University of Toronto Toronto Ontario Canada; ^2^ Centre for Global Child Health Hospital for Sick Children Toronto Ontario Canada; ^3^ Parkdale Queen West Community Health Centre Toronto Ontario Canada; ^4^ Department of Anthropology University of Toronto Toronto Ontario Canada; ^5^ Translational Medicine Hospital for Sick Children Toronto Ontario Canada; ^6^ Department of Paediatrics Mount Sinai Hospital Toronto Ontario Canada

**Keywords:** child health, community participation, Delphi technique, public health, maternal health, Nutrition Programs, perinatal care

## Abstract

The Canada Prenatal Nutrition Program (CPNP) is a federal initiative that addresses the critical impact of early nutrition and perinatal care on health outcomes by providing community‐based perinatal health and nutrition services to socially and economically vulnerable women and their infants. Using a flexible, locally adaptable community programming model, the CPNP aims to improve the health of women and their babies by promoting healthy birth weights and supporting breastfeeding; fostering community partnerships; and providing culturally sensitive support. To identify priorities for future research and outcome measurement within the program's monitoring and evaluation framework, we engaged CPNP personnel from 26 sites across the province of Ontario as expert knowledge holders in a Modified Delphi study. Among participating sites, 57% were hosted within a community‐based agency, 23% within an Indigenous‐led agency, and 20% within a public health unit. Participants ranked priorities based on what they believed best reflected CPNP clients' needs, contributed to evidence‐based program development, and aligned with the realities of community‐based service delivery. All priorities were viewed as important for programming. From the relative rankings, two research topics were highest‐ranked: assessing the impacts of the CPNP and conducting a needs assessment to identify community‐defined perinatal health needs. Maternal mental health was the highest‐ranked outcome measure, because of its perceived central role in supporting parent–infant health, well‐being, and social connectedness. Collectively, these applied findings offer strategic direction to advance the CPNP's adaptive program delivery model, and demonstrate the methodological value of engaging community knowledge holders to inform perinatal program delivery.

## Key Points

- CPNP personnel maintain trusted relationships with vulnerable mothers and infants, highlighting their frontline insight to improve programming effectiveness, equity, and reach.
- They prioritized research topics on health outcomes to inform program design and demonstrate the program's value.
- The top‐ranked outcome measure for monitoring and evaluation was maternal mental health, given its relevance to perinatal health, well‐being, and programming.
- Increasing complexity in social determinants of health faced among CPNP clients, stagnant funding, and escalating operational costs increasingly constrained CPNP programs' capacity to sustain service frequency and quality.
- Community stakeholder involvement in priority setting can inform perinatal program development across diverse contexts.

## 1 Introduction

Investment in perinatal health and nutrition during the first 1000 days of life is critical for promoting lifelong health and development (UNICEF 2020). Over the past two decades, Canada has consistently ranked in the lower half of high‐income countries on UNICEF's measures of overall child well‐being, including physical health and survival (UNICEF Canada 2020, 2025). Child poverty and food insecurity are noted to be growing nationally, with vulnerable Canadians facing increasingly complex social determinants of health—particularly along socioeconomic and ethno‐racial lines (Campaign 2000, 2024). There is a call for improved social safety nets to protect and support the health of children at risk within the Canadian population (Children First Canada 2025; UNICEF Canada 2025).

The Canada Prenatal Nutrition Program (CPNP) is a nationally implemented approach to supporting high‐risk families within the first 1000 days through community health programming. Announced in 1994 as part of Canada's commitment to the *United Nations Convention on the Rights of the Child*, the CPNP is federally funded by the Public Health Agency of Canada to support pregnant and postnatal women and their infants facing multiple risk factors (e.g., poverty, social isolation, recent immigration, Indigenous identity) (Public Health Agency of Canada 2025; Schewndt 2002). Rooted in a population health approach, the CPNP aims to improve maternal and infant health by increasing healthy birth weights and promoting and supporting breastfeeding through culturally‐sensitive and accessible programming delivered via partnerships and collaborations within communities (Public Health Agency of Canada 2025). Targeted to the needs of the communities they serve, CPNP sites offer different supports, such as nutrition education, breastfeeding support, food supplementation, perinatal health counseling, and/or referrals to health and social services.

The CPNP funding allocation has remained unchanged since the 1990s, despite the program's perceived benefits among clients, demonstrated reach to target populations, economic value, and suggested impact on health outcomes (McMurchy et al. 2009; Muhajarine et al. 2012; Public Health Agency of Canada 2018). Currently, there is minimal programmatic guidance or routine monitoring and evaluation to inform CPNP program implementation. With the rate of inflation and an increasing number of Canadians experiencing intersecting barriers related to the social determinants of health (e.g., mental health needs, food insecurity, housing instability), reflecting on multiple components of the CPNP could help improve programmatic delivery (Baxter et al. 2024).

In the context of rising inequities nationwide, the growing gaps between child well‐being, evolving community needs, and CPNP funding are cause for concern. Community stakeholders, such as CPNP lead personnel, are often under‐represented in research to inform program delivery and guide evaluation, despite their central position as expert knowledge holders in delivering community‐level programming. This study employed a community‐engaged, participatory Modified Delphi process to generate consensus and establish priorities for future research topics and outcome measures for monitoring and evaluation within the CPNP. By engaging program delivery personnel, this study generates a basis for practice‐informed research and evaluation that can inform future program implementation and assessment.

## 2 Methods

This study was conducted as part of a larger program of CPNP‐engaged research. Initial studies investigated infant feeding practices, lactation support delivery, and household food security at specific sites (Francis et al. 2021; Francis et al. 2020; Mildon et al. 2023). Building from this work, we aim to inform program enhancements across the wider CPNP from an implementation perspective. To determine priorities for future research topics and outcomes for monitoring and evaluation, we employed a Modified Delphi process, incorporating elements of the Nominal Group Technique, to systematically gather and prioritize expert input (Rankin et al. 2016). The “Delphi studies in social and health sciences–recommendations for an interdisciplinary standardized reporting” (DELPHISTAR) checklist was followed in reporting this study (Niederberger et al. 2024).

### 2.1 Participant Identification and Research Events

To capture diverse representation, this study engaged CPNP sites across the province of Ontario in 1‐day research events held in five regions: Northeast, Northwest, East, Central, and Southwest. Eligible participants included English‐speaking CPNP site coordinators, or a delegate with similar qualifications (e.g., coordinating and/or leading CPNP programming at a CPNP site) in Ontario. Because this was a program‐level assessment, we focused on CPNP site personnel's perspectives given their direct engagement with clients and extended tenure within the program, making them well positioned as key informants.

Sites were identified from a previously conducted national survey of CPNP programming (Baxter et al. 2024), in which 33 sites in Ontario self‐indicated their willingness to take part in further research. These sites were contacted via email, and 22 (67%) joined the study. For the Northern regions, recruitment also included word of mouth and an advertisement within the provincial CPNP network, which led to communication with four additional sites, all of which joined the study. In total, 26 sites were engaged (Supporting Information S1: Figure 1). All communication and events were conducted in English. In each region, the 1‐day research events were hosted by a participant's CPNP site, except in the Northeast where a hotel was used. Due to weather conditions, the events in the Northern regions were conducted in a hybrid format, while the other events were conducted in‐person.

**Figure 1:**
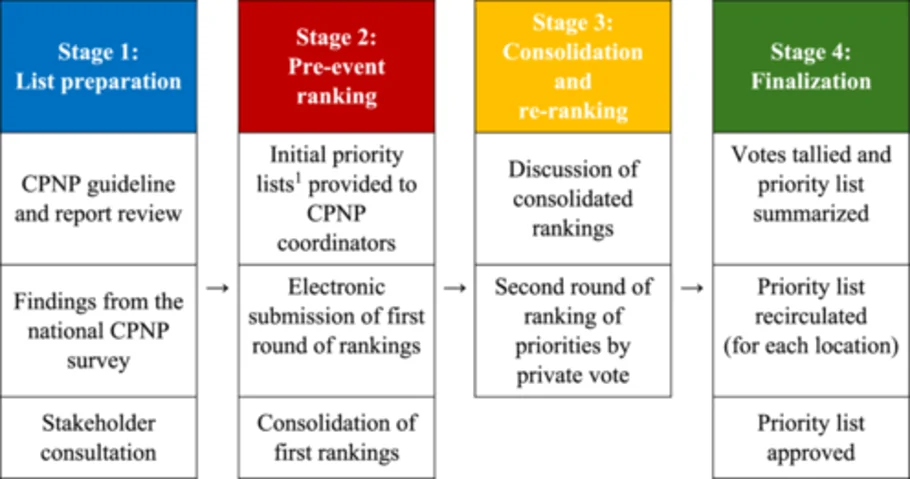
Modified Delphi process progression of events^†^. ^†^Two lists were provided to participants to rank: perinatal nutrition and health (1) future research topics and (2) outcome measures for monitoring and evaluation.

### 2.2 Sample Size

We aimed to recruit 6–8 participants per region, and a maximum of 32 participants. Because the 1‐day research events also included a focus group discussion exercise, the sample size was based on standard focus group discussion methodology, which suggests enrolling 6‐8 participants per group (Krueger 2002). Hosting events with groups of this size allowed for a range of opinions, experiences, and ideas to facilitate discussion.

### 2.3 Participant and Site Information

At each event, participants were asked to complete a paper‐based demographic form that included items on the geographic location of their CPNP site (large population center, medium population center, small population center, rural area); how long they had worked there (years); if they had worked at another CPNP site before (yes/no) and how long; and to rank the top three sociodemographic profiles of clients accessing their site (low‐income households, lone parents, recent immigrants [i.e., living in Canada < 10 years], Indigenous identity, low education [i.e., < high school], teenage pregnancy).

### 2.4 Delphi Consensus‐Gathering Process

Employing a Modified Delphi process, with elements of Nominal Group Technique (Rankin et al. 2016), our approach combined iterative rounds of feedback with structured group discussion to enhance consensus generation and ensure stakeholder engagement, and took place over four stages (Figure 1). All participants completed all study stages.

#### 2.4.1 Stage 1: List Preparation

In advance of meeting with participants, two lists of possible perinatal health and nutrition research topics and outcome measures for monitoring and evaluation were generated by the lead researchers (JABB and AM). Item selection was guided by the aims and operating principles of the CPNP (Canada Prenatal Nutrition Program, First Nations and Inuit Health Branch, & Health Canada 2009; Public Health Agency of Canada 2007); review of CPNP reports (Health Canada, & Public Health Agency of Canada 2016; Office of Audit and Evaluation, Health Canada, & Public Health Agency of Canada 2021); and findings from the national survey of CPNP lactation and food programming (Baxter et al. 2024). Eleven items were identified for each list, and some items included examples to improve clarity (Supporting Information S1: Tables 1 and 2). Given the diversity of programming between CPNP sites, both lists were reviewed and discussed with a small group of CPNP coordinators based in Ontario (*n* = 3) who had previously volunteered to serve on the Advisory Group for the national survey. The lists were finalized based on this input.

#### 2.4.2 Stage 2: Pre‐Event Ranking

Participants were provided with both lists 2 weeks in advance of their regional event. The lists were shared using the online platform Research Electronic Data Capture (REDCap; Vanderbilt University, TN). Based on the assumption of adequate resourcing being available to support the list activities, participants individually ranked the items on each list in priority order (1 being highest priority, 11 being lowest priority). Participants were also invited to suggest any additional future research topics and outcome measures for monitoring and evaluation not listed.

#### 2.4.3 Stage 3: Consolidation and Re‐Ranking

Prior to each research event, the pre‐ranking future research topics and outcome measures for monitoring and evaluation results were collated, synthesized, and used to inform the presentation of the list items. Rank order was determined by mean for each list. The mean and range of ranking for each item were shared with participants and discussed using Nominal Group Technique for consensus development (Rankin et al. 2016). Participants were invited to consider whether any priorities could be consolidated, added, or removed. JABB led the discussions, which were approximately 90 min, and AM took detailed notes. Care was taken to ensure all participants' voices were considered and captured. Following discussion, each participant confidentially re‐ranked each list on a paper form. Immediately following each research event, JABB and AM further conferred about the discussion and added to the notes, including any clarification of terms and reflection on reasons for prioritizing different items in the lists.

#### 2.4.4 Stage 4: Finalization

Following each research event, individual future research topic and outcome measure for monitoring and evaluation priority rankings from Stage 3 were compiled and summarized for each list. The results (pre‐ranking, re‐ranking, and top five priorities) were shared via email to participants. Participants were asked to confirm their agreement with the results and whether they felt consensus had been achieved.

### 2.5 Data Analysis

Data from the paper‐based demographic form and priority setting exercises were entered into Excel (Microsoft Corporation, Redmond, WA), and exported into Stata, version 15.0 (Stata Corporation, College Station, TX). Descriptive statistics were used to describe participants characteristics and their CPNP sites, including means and standard deviations (SD) and proportions for categorical variables. For the pre‐ranking data (Stage 2), the mean ranking and range for each item in each list were shared and discussed with participants at the research events (Stage 3) and in the finalization stage (Stage 4). Data were pooled from all research events after confirming there was consistency on the prioritized future research topics and outcome measures for monitoring and evaluation across the five research events. To summarize data from each list, mean and SD and median and interquartile range (IQR) were used.

### 2.6 Ethics Statement

Prior to recruitment, ethical approval was obtained from research ethics boards at the Hospital for Sick Children (REB number: 1000080620) and University of Toronto (Protocol #: 00045511). Written, informed consent was provided by all participants. Participants received a $50 gift card as a token of appreciation, their travel costs to attend the research events were compensated, and lunch and refreshments were provided.

## 3 Results

Of the 37 sites contacted for participation, 2 sites did not reply and 9 sites were unable to participate (7 unavailable, 2 transportation concerns). Twenty‐six sites participated, including 28 individual participants. Five research events took place between January to April 2024, including the Northeast (Timmins [*n* = 5 participants; 4 sites]), Northwest (Thunder Bay [*n* = 7 participants; 6 sites]), East (Kingston [*n* = 6 participants; 6 sites]), Central (Toronto [*n* = 7 participants; 7 sites]), and Southwest regions (Hamilton [*n* = 3 participants; 3 sites]).

Ten of the participating CPNP sites (38%) were based in large, urban centers, eight (31%) in medium population centers, and four each in small population centers and rural areas (15%, respectively). Fifty‐seven percent of sites were hosted within a community‐based agency, 23% within an Indigenous‐led agency, and 20% within a public health unit. Most (54%) of the participants had worked with the CPNP for at least 5 years (mean ± SD: 9 ± 8 years). CPNP sites reported serving clients from all priority sociodemographic groups: low‐income households (26 sites [100%]), recent immigrants (15 sites [58%]), lone parents (12 sites [46%]), low education (11 sites [42%]), Indigenous identity (10 sites [38%]), and teenage pregnancy (5 sites [19%]) were as the three main sociodemographic groups served.

### 3.1 Rankings Within the Modified Delphi Process

Rankings from the Modified Delphi process reflect relative prioritization (Tables 1 and 2). While the highest‐ranked items are distinguishable from lower rankings, overlapping variability indicates that several closely ranked items may represent similarly high‐priority areas.

### 3.2 Ranking of Future Research Topics

After the final ranking (Stage 3), the average rank order of future research topics ranged from 4.5 (highest ranking) to 7.5 (lowest ranking), with overlapping distributions across items (Table 1). Participants noted that all the future research topics were highly relevant to the CPNP. Throughout the discussion, participants repeatedly expressed interest in research on understanding clients' needs and well‐being, rather than their own in their role.

**Table 1:** Results for future research topic priorities among Ontario‐based CPNP lead personnel (*n* = 28), determined using a Modified Delphi process with Nominal Group Technique to generate consensus.

Item	Pre‐event ranking	Final ranking	Final ranking mean (SD)^a^	Final ranking median (IQR)
Evaluate the impacts of CPNP programming on perinatal health and well‐being outcomes	1	1	4.5 (3.2)	4.5 (1, 6.5)
Needs assessment to understand community priorities related to perinatal/family health and well‐being	2	1	4.5 (3.2)	4 (1.5, 7)
Understand and document best practices for program delivery	7	3	5.8 (2.6)	5 (3.5, 7.5)
Explore CPNP participants' experiences and perceptions of the program	3	4	5.9 (2.9)	6 (3, 8)
Engage CPNP participants to develop and test new programming activities or resource materials	4	5	6.0 (3.1)	6 (3, 9)
Follow up CPNP participants to learn about longer‐term impacts of programming on maternal and infant health and well‐being outcomes	5	6	6.1 (2.7)	6 (4, 8)
Investigate how the social determinants of health affect perinatal health and well‐being outcomes	6	7	6.2 (3.7)	6 (3, 10)
Cost‐benefit analysis of CPNP programming	11	8	6.3 (3.1)	7 (3.5, 8)
Pilot test innovative programming activities	9	9	6.4 (3.6)	7 (2, 9.5)
Document stories of CPNP participants' lived experiences and the impact of the CPNP	8	10	6.8 (2.9)	7 (4, 9)
Explore CPNP coordinators' experiences and perceptions of their role and the factors that enable them to lead impactful programming	10	11	7.5 (3.0)	9 (4.5, 10)

Abbreviations: IQR, Interquartile range; SD, Standard deviation.

^a^ Priority order: 1 being highest priority, 11 being lowest priority

Two items received the highest relative ranking among the future research topics during both the pre‐ranking (Stage 2) and re‐ranking stages (Stage 3): evaluating the impacts of CPNP programming on perinatal health and well‐being outcomes; and needs assessment to understand community priorities related to perinatal/family health and well‐being (Table 1). Both items related to supporting evidence‐based program design, and the mean ± SD were 4.5 ± 3.2 (Table 1); the median (IQR) for evaluating the impact of CPNP on outcomes and conducting a needs assessment were 4.5 (1, 6.5) and 4 (1.5, 7), respectively. The lowest relative ranking among the research topics related to exploring CPNP coordinators' experiences and perceptions of their role and the factors that enable them to lead impactful programming. For this item, the mean ± SD was 7.5 ± 3.0, with a median (IQR) of 9 (4.5, 10).

### 3.3 Ranking of Outcome Measures for Monitoring and Evaluation

For outcome measures for monitoring and evaluation, the average rank order ranged from 3.3 (highest) to 8.9 (lowest) following the final ranking, and distributions overlapped for some items (Stage 3; Table 2). Across events, participants noted interest in measuring outcomes that underlie perinatal health and well‐being and reflected the supports that their sites actually offered. The top five outcome measures for monitoring and evaluation were maintained between the pre‐event ranking and re‐ranking stages, and all listed items were considered important to the CPNP.

**Table 2:** Results for outcome measures for monitoring and evaluation priorities among Ontario‐based CPNP lead personnel (*n* = 28), determined using a Modified Delphi process with Nominal Group Technique to generate consensus.

Item	Pre‐event ranking	Final ranking	Final ranking mean (SD)^a^	Final ranking median (IQR)
Maternal mental health	1	1	3.3 (2.9)	2.5 (1, 4)
Social support/community connectedness	3	2	4.3 (2.4)	4 (2, 6)
Parent‐infant attachment	2	3	4.4 (2.7)	4 (2, 6.5)
Maternal nutrition	5	4	4.7 (2.5)	4 (3, 6.5)
Infant feeding	4	5	4.9 (2.9)	4 (3, 8)
Life skills, food access, and food literacy	8	6	5.9 (2.8)	5 (4, 7.5)
Knowledge about perinatal health and nutrition	6	7	6.6 (2.5)	7 (5.5, 8)
Pregnancy health	7	8	7.5 (2.8)	8 (7, 9)
Parenting self‐confidence	10	9	7.9 (2.4)	8 (6, 10)
Substance use	11	10	8.7 (2.3)	9 (7, 11)
Infant size at birth	9	11	8.9 (2.9)	10 (6.5, 11)

Abbreviations: IQR, interquartile range; SD, standard deviation.

^a^ Priority order: 1 being highest priority, 11 being lowest priority

Maternal mental health received the highest relative ranking among the outcomes to measure, due to its perceived effect on multiple aspects of perinatal well‐being (Table 2). For this item, the mean ± SD was 3.3 ± 2.9, with a median (IQR) of 2.5 (1, 4). Substance use and infant size at birth received the lowest relative ranking (8.7 ± 2.3 and 8.9 ± 2.9, respectively). For infant size at birth, it was noted that other health‐factors outside of CPNP programming affect infant growth (e.g., gestational diabetes) and that mothers may not attend CPNP programming until later postpartum, limiting their exposure to programming.

## 4 Discussion

This priority setting exercise engaged CPNP personnel and used a consensus technique to identify research topics and outcome measures for monitoring and evaluation for CPNP programming in Ontario. In ranking both lists, participants based their decisions on what they believed was in CPNP clients' best interests, contributed to evidence‐based program design, and could be influenced by the community‐embedded programming implemented at CPNP sites. Two future research topics were ranked highest: evaluating the impacts of CPNP programming on perinatal health and well‐being outcomes; and conducting needs assessments to understand community priorities related to perinatal/family health and well‐being. Maternal mental health was ranked the highest among the outcome measures for monitoring and evaluation. Importantly, this study demonstrates how a community‐engaged priority setting exercise can generate research and evaluation priorities that reflect the perspectives of those involved in program implementation.

### 4.1 Interpretive Lens and Positioning Employed by Participants During Ranking Exercises

Across research events, participants recognized that all of the presented research topics and outcome measures for monitoring and evaluation were very important and relevant to the CPNP. This made ranking the listed items difficult and was reflected in the overlap between SDs and IQRs. Additionally, stagnant CPNP funding presents a significant challenge to delivering the CPNP within communities, and this has been exacerbated since the pandemic, as CPNP clients experience increased vulnerabilities relative to the 1990s (Baxter et al. 2024; Office of Audit and Evaluation et al. 2021). This context was at the forefront of the ranking exercise discussions, as participants considered which items would best reflect the current realities of program delivery and most effectively convey the CPNP's value and strengths to the funder (the Public Health Agency of Canada). Of note, this interpretive lens emerged independently and consistently in the research events.

### 4.2 Contextualizing Top Relative Rankings for Future Research Topics as Identified and Ranked by Participants

Participants initially found it challenging to conceptualize how their sites might engage in research, given their heightened awareness of funding limitations and that they are often at capacity completing their day‐to‐day programmatic responsibilities. However, reiterating that the ranking exercise assumed that funding and appropriate supports would be in place led to enthusiasm for the higher ranking of items perceived to strengthen CPNP programming. With respect to the research topic on *evaluating the impacts of CPNP programming on perinatal health and well‐being outcomes*, participants felt this was important not only for demonstrating the CPNP's value, but also for informing evidence‐based programming approaches to support clients. To date, studies investigating the impact of different CPNP programming models on perinatal outcomes have not been conducted. A longitudinal study across multiple CPNP sites delivering varying levels of program intensity had been proposed in the early 2000s to compare the effects and cost‐effectiveness, but did not receive funding (PRA Inc. Information Info Strategy 2004). Evaluating the impact of different program designs on perinatal health and well‐being outcomes would offer an opportunity to strengthen evidence‐informed programming within the CPNP, as has been recognized by the funder (Office of Audit and Evaluation et al. 2021). Regarding the research topic to *conduct a needs assessment to understand community priorities related to perinatal/family health and well‐being*, this likely reflected CPNP sites' effort to deliver contextualized programming amidst the challenges they face. While CPNP sites in Ontario are generally considered to reach their target populations (Public Health Agency of Canada 2018), developing tools and resources to support needs assessment conduction could further enhance their capacity to deliver community‐embedded programming. The identification of this research topic also likely reflected study timing. At data collection, CPNP sites were undergoing a renewal process with the funder, requiring a needs assessment. Given CPNP sites' variation in skill sets and resources, support to conduct such assessments was perceived as highly beneficial for communicating with the funder.

### 4.3 Contextualizing Relative Rankings Among Outcomes for Monitoring and Evaluation as Identified and Ranked by Participants

Since its inception, the CPNP has included a national evaluation framework with objectives, evaluation tools, and indicators (Schewndt 2002). Historically, these indicators have included both impact‐related outcomes (e.g., individual health outcomes) and processes‐oriented measures (e.g., program development, target population reach, implementation). However, the depth (e.g., number of indicators) and manner of collection (e.g., individual participant data, program‐level data) have evolved over time, including the discontinuation of comprehensive participant‐level birth data collection in 2006 (Muhajarine et al. 2009; Schewndt 2002). Current reporting focuses on program and participation characteristics; target population reach; and self‐reported participant experiences, health behaviors, and knowledge (Public Health Agency of Canada 2018). There is a need to re‐invest in outcome assessment, and the findings from this study could be leveraged to inform a more complete program impact pathway. While all listed outcomes for monitoring and evaluation were recognized as very important to CPNP programming, those outcomes CPNP personnel prioritized tended to align with areas where they perceived their programming had influence and impact (i.e., maternal mental health, social support and community connectedness, parent‐infant attachment), rather than health measures and behaviors that can be influenced by complex external factors (i.e., infant size at birth, substance use).

Maternal mental health, the outcome with the highest relative ranking, represents a global measure of perinatal well‐being that underpins other outcomes, and aligns well with the CPNP logic model ultimate outcome (“Canadians have improved physical and mental health”) (Office of Audit and Evaluation et al. 2021). From a biological perspective, there are known relationships between maternal mental health and breastfeeding and low birthweight, respectively (Arabzadeh et al. 2024; Butler et al. 2021; Dennis and McQueen 2009; Grote et al. 2010). In the general population, approximately 20% of women report experiencing perinatal anxiety (Dennis et al. 2017), and 23% of mothers in Canada report feelings consistent with postpartum depression or an anxiety disorder (Statistics Canada 2019). In the most recent CPNP client survey, 84% of mothers reported stress—a critical determinant of maternal mental health and overall perinatal well‐being—as a challenge they faced (Public Health Agency of Canada 2018). Participants repeatedly emphasized how poor maternal mental health had a cascading effect on other outcomes and was foundational to parent‐infant health, well‐being, and social connectedness. Poor perinatal mental health is also associated with adverse developmental outcomes (Rogers et al. 2020), although this can be mediated by parenting quality and social support (Stein et al. 2014). Notably, social determinants of health increase mental health risks (Lund et al. 2018). Prioritization of maternal mental health reflected its essential role in achieving broader program goals, thus reflecting the relational, community‐embedded model of the CPNP. This finding supports broadening evaluation frameworks to recognize maternal mental health as an important outcome and as a potential contributor to longer‐term maternal and infant health.

Substance use and infant size at birth had the lowest relative ranking from the list of priority outcomes. Nationally, compared to other life challenges, fewer CPNP clients reported substance use as a challenge (25% reported smoking, 7.5% drinking alcohol, and 9% drug/alcohol use in the family) (Public Health Agency of Canada 2018), which could have led to its de‐emphasis by CPNP personnel. This may reflect a decline in the prevalence of substance use compared to the 1990s and a shift away from the original CPNP target populations (Public Health Agency of Canada 2018). Within Ontario, there has been a 46% decrease in prenatal tobacco use between 2012 and 2022, from 11% to 6%, although prenatal alcohol use has remained at 3% (Pratt Tremblay et al. 2025). There was discordance between participants' overall ranking of infant size at birth as the lowest relative ranking among outcome measures for monitoring and evaluation, yet “increasing the number of babies born at a healthy weight” is a stated primary goal of the CPNP (Public Health Agency of Canada 2025). Some participants identified that clients present to programming late in their pregnancy and/or are affected by factors external to program reach (e.g., comorbidities, pre‐existing biological factors), thus they did not feel that infant size at birth as an outcome reflected what current CPNP programming could most tangibly affect, especially in the context of static funding, rising operational costs, and increasingly complex client needs (Office of Audit and Evaluation et al. 2021). This points to a mismatch between the funder‐defined outcomes and those valued by community implementers, and highlights the importance of measuring both program implementation and short‐ and long‐term health outcomes within an evaluation framework.

### 4.4 Implications for Policy

An important implication of our findings is the potential tension between funder‐defined and coordinator‐defined measures of program success. Funders often prioritize biological health outcomes to demonstrate effectiveness, whereas the coordinators in this study saw the importance of these outcomes but also emphasized a broader range of participant well‐being outcomes, including social support and service accessibility. Rather than being competing priorities, these perspectives reflect complementary dimensions of program effectiveness. Evaluation frameworks that balance biological, social, and implementation outcomes may provide a more comprehensive assessment of complex community‐based interventions.

### 4.5 Engaging CPNP Personnel as Expert Knowledge Holders and a Critical Planning Asset

As demonstrated by the high enrollment rate, CPNP personnel were eager to engage in this research to inform CPNP programming. The inclusion of CPNP personnel has been recognized by the funder as important to supporting health and well‐being and optimizing CPNP reach (Office of Audit and Evaluation et al. 2021). Within the most recent national CPNP client survey, the majority of clients noted having had a positive and supportive CPNP experience: 96% reported feeling welcomed and accepted; 96% noted personnel responded to their concerns; 95% suggested their personal and cultural beliefs were respected; and 95% reported personnel helped them learn how to get to the resources they needed (Public Health Agency of Canada 2018). This speaks highly to the importance of engaging CPNP personnel as expert knowledge holders, given their one‐on‐one and valued interactions with clients. The continued engagement of CPNP personnel in decision‐making would be an asset to strengthening the program.

### 4.6 Strengths and Limitations

A strength of this study was that diverse regions of Ontario and CPNP sites (e.g., agency type, locality) were engaged. However, some invited CPNP sites were at capacity or short‐staffed and unable to participate, and recruitment was largely drawn from the previously conducted survey. Due to the representativeness of the top three client groups reported by participants, we do believe those sites that participated in the study reflected the broad demographics of Ontario CPNP sites. Employing a Modified Delphi process was a strength of the study, as it recognized the insight of CPNP personnel as experts in their context. Although the top priorities were consistent between the pre‐ and re‐ranking phases, conducting a third round of ranking that included only the top five items in each list could have further reduced some of the spread in responses. We did not engage CPNP participants, the funder, or other community members in this study, which may have led to the identification of different priorities. While Ontario has the largest proportion of CPNP sites nationally (approximately 35%), it is possible that priorities may differ relative to other regions of Canada. This method could be adapted for use in other provincial or international maternal and child health programs seeking to align research and evaluation priorities with the perspectives of program delivery stakeholders. At one research event, it was noted that conducting the priority setting exercises, or a similar process, with Indigenous organizations only could be of benefit as the priorities identified may differ. Reflection on the present findings and further engagement of Indigenous communities should be considered.

### 4.7 Implications

This study demonstrated CPNP personnel's commitment to strengthening community‐based programming in a manner that aligns with both client needs and operational realities. After three decades of implementation, these findings offer valuable insights to inform the next phase of the CPNP's flexible delivery model, as well as the development and enhancement of relevant monitoring and evaluation frameworks. To understand health outcomes over time, linkages with health administrative data could be considered. This study highlights the broader value of engaging community‐level implementers as expert knowledge holders, offering lessons for the development of perinatal programs beyond the Canadian context. Continued engagement with CPNP personnel should be a priority to support the co‐design of any future research and evaluation initiatives.

## Author Contributions

J.A.B.B. and A.M. conceptualized this study, secured funding, designed and conducted the research, and interpreted the findings. D.L.O., D.W.S., and B.U. provided feedback on the research question, design, framing, and interpretation. J.A.B.B. analyzed the data and produced the first manuscript draft, with input from A.M. All authors critically reviewed the manuscript and approved the final version.

## Conflicts of Interest

The authors declare no conflicts of interest.

## Supporting information


Supporting File

## Data Availability

Deidentified individual participant data (including data dictionaries), analytic code, and study protocol will be made available to researchers who submit a methodologically sound proposal and following human research ethics approval. Requests should be directed to the corresponding author and will be reviewed to ensure alignment with the aims of the approved proposal.
