# Supplementary material for: Research and Outcome Priorities for the Canada Prenatal Nutrition Program in Ontario: A Community‐Engaged Modified Delphi Study
Source: Matern Child Nutr. 2026 Oct 15;22(4):e70257. doi: 10.1111/mcn.70257 (PMC13670009; doi:10.1111/mcn.70257)
Supplement: Supplementary file 1 [file MCN-22-e70257-s001.docx]

**APPENDICES**

|  |  | CPNP sites that completed national survey (n=91) | |  |  |
| --- | --- | --- | --- | --- | --- |
|  |  |  |  |  |  |
|  |  |  |  |  | Site not in Ontario (n=51)  Declined further contact for research purposes (n=6)  Non-English speaking (n=1) |
|  |  |  |  |  |  |
|  |  | English-speaking CPNP sites in province of Ontario eligible to participate in study (n=33) | |  |  |
|  |  |  |  |  |  |
| Additional northern CPNP sites identified by word of mouth (n=4) |  |  |  |  | Declined participation (n=9)  Unavailable (n=7)  Transit concern (n=2)  Unable to contact (n=2) |
|  |  |  |  |  |  |
|  |  | CPNP sites enrolled in study (n=26) | |  |  |
|  |  |  |  |  |  |

**Supplemental Figure 1**. Flow diagram of sites eligible for and enrolled in the CPNP Modified Delphi study

**Supplemental Table 1.** List of potential future CPNP research topics for ranking

| **Research topic** | **Explanation or Examples** |
| --- | --- |
| Evaluate the impacts of CPNP programming on perinatal health and well-being outcomes |  |
| Follow up CPNP participants to learn about longer-term impacts of programming on maternal and infant health and well-being outcomes |  |
| Explore CPNP participants’ experiences and perceptions of the program | Focus group discussions about the services provided at CPNP sites by the host agency and partners |
| Explore CPNP coordinators’ experiences and perceptions of their role and the factors that enable them to lead impactful programming |  |
| Needs assessment to understand community priorities related to perinatal/family health and well-being |  |
| Investigate how the social determinants of health affect perinatal health and well-being outcomes |  |
| Understand and document best practices for program delivery | Knowledge exchange between CPNP sites |
| Pilot test innovative programming activities |  |
| Cost-benefit analysis of CPNP programming |  |
| Engage CPNP participants to develop and test new programming activities or resource materials | Collaborative design process |
| Document stories of CPNP participants’ lived experiences and the impact of the CPNP | Digital storytelling project |

**Supplemental Table 2.** List of potential CPNP outcome measures for monitoring and evaluation for ranking

| **Outcome** | **Explanation or Examples** |
| --- | --- |
| Infant size at birth | Birthweight, gestational age, preterm birth |
| Infant feeding | Breastfeeding initiation, duration, exclusivity; introduction of solids |
| Parent-infant attachment | The bond between a parent and their infant/s |
| Maternal nutrition | Dietary intake, prenatal supplement use, etc. |
| Pregnancy health | Gestational weight gain, physical activity, gestational diabetes, etc. |
| Maternal mental health | Stress, anxiety, depression, trauma |
| Substance use | Smoking/alcohol/cannabis/drug/combined substance use during pregnancy and/or lactation |
| Life skills, food access, and food literacy | skills, understanding food labels |
| Social support/community connectedness | Access to providers, services and supports relevant to participant needs |
| Parenting self-confidence |  |
| Knowledge about perinatal health and nutrition | Healthy eating, self-care, infant care, etc. |
